# Pyriproxyfen for mosquito control: female sterilization or horizontal transfer to oviposition substrates by *Anopheles gambiae* sensu stricto and *Culex quinquefasciatus*

**DOI:** 10.1186/1756-3305-7-280

**Published:** 2014-06-21

**Authors:** Oscar Mbare, Steven W Lindsay, Ulrike Fillinger

**Affiliations:** 1 International Centre of Insect Physiology and Ecology (icipe), Thomas Odhiambo Campus, 40305 Mbita, Kenya; 2 Disease Control Department, London School of Hygiene & Tropical Medicine, WC1E 7HT London, United Kingdom; 3 School of Biological and Biomedical Sciences, Durham University, DH1 3LE Durham, United Kingdom

**Keywords:** *Anopheles gambiae*, *Culex quinquefasciatus*, Vector control, Insect growth regulator, Pyriproxyfen, Sterilization, Auto-dissemination

## Abstract

**Background:**

The use of gravid mosquitoes as vehicles to auto-disseminate larvicides was recently demonstrated for the transfer of pyriproxyfen (PPF) by container-breeding *Aedes* mosquitoes and presents an appealing idea to explore for other disease vectors. The success of this approach depends on the female’s behaviour, the time of exposure and the amount of PPF that can be carried by an individual. We explore the effect of PPF exposure at seven time points around blood feeding on individual *Anopheles gambiae* sensu stricto and *Culex quinquefasciatus* fecundity and ability to transfer in laboratory assays.

**Method:**

Mosquitoes were exposed to 2.6 mg PPF per m^2^ at 48, 24 and 0.5 hours before and after a blood meal and on the day of egg-laying. The proportion of exposed females (N = 80-100) laying eggs, the number of eggs laid and hatched was studied. Transfer of PPF to oviposition cups was assessed by introducing 10 late instar insectary-reared *An. gambiae* s.s. larvae into all the cups and monitored for adult emergence inhibition.

**Results:**

Exposure to PPF between 24 hours before and after a blood meal had significant sterilizing effects: females of both species were 6 times less likely (Odds ratio (OR) 0.16, 95% confidence interval (CI) 0.10-0.26) to lay eggs than unexposed females*.* Of the few eggs laid, the odds of an egg hatching was reduced 17 times (OR 0.06, 95% CI 0.04-0.08) in *Anopheles* but only 1.2 times (OR 0.82, 95% CI 0.73-0.93) in *Culex*. Adult emergence inhibition from larvae introduced in the oviposition cups was observed only from cups in which eggs were laid. When females were exposed to PPF close to egg laying they transferred enough PPF to reduce emergence by 65-71% (95% CI 62-74%).

**Conclusion:**

PPF exposure within a day before and after blood feeding affects egg-development in *An. gambiae* s.s. and *Cx. quinquefasciatus* and presents a promising opportunity for integrated control of vectors and nuisance mosquitoes. However, sterilized females are unlikely to visit an oviposition site and therefore do not transfer lethal concentrations of PPF to aquatic habitats. This suggests that for successful auto-dissemination the optimum contamination time is close to oviposition.

## Background

Mosquito larval source management is an effective method for controlling mosquito-borne diseases [1-5]. However, application of larvicides requires labour intensive programmes that are complex to organize and expensive to run [6-8]. Thus novel strategies for larvicide application need to be explored to minimize efforts and costs [9,10]. Using the gravid female mosquito as a vehicle to auto-disseminate larvicides has been demonstrated recently for the transfer of pyriproxyfen (PPF) by container-breeding *Aedes* mosquitoes [11-13] and presents an appealing idea to explore for the control of other mosquito genera.

PPF is a juvenile hormone mimic and affects immature and adult mosquito stages in different ways [14-17]. The major effect of PPF on mosquitoes is the inhibition of metamorphosis to prevent emergence of adults from pupae [18,19]. PPF has extremely low toxicity to humans [20], is effective at controlling mosquito larvae at very low doses [16,21] and can persist for up to six months in a variety of aquatic habitat types [15,21-23]. In addition, exposure of larvae to sub-lethal doses of PPF affects the adults’ egg development, egg production and reduces the hatching of eggs [24,25]. Exposure to PPF has been studied extensively in *Aedes* mosquitoes [11,13,15,26-28] and it has been shown that topical application can also reduce the reproductive capacity of adults [13,15,27,28] depending on dosage and time of exposure in relation to the blood meal [13], which signals the start of egg development [29]. However, inconsistent information on the effect of PPF exposure on egg-laying and hatching of eggs laid can be found for various species requiring more research in this subject area [13,15,30,31].

To date only three studies have evaluated the impact of topical contact of PPF on *Anopheles gambiae sensu* lato, the major African malaria vector [17,32,33]. Ohashi and colleagues [32] exposed *An. gambiae* s.s. to treated nets immediately before or after a blood meal and reported complete sterilization in females exposed to nets that retained an approximate dose of 35 mg/m^2^ PPF and 3.5 mg/m^2^ PPF. However, at a 10 times lower dosage the proportion of females laying eggs was reduced by less than 50% compared to the control when exposed just before the blood-meal and not at all when exposed after the blood meal. A more recent study by Ngufor and colleagues [33] confirmed complete sterilization in wild *An. gambiae* s.s. exposed to PPF-treated nets in experimental hut trials. Harris and colleagues [17] however, observed complete sterilization of female *An. arabiensis* only 24 hours after the bloodmeal (exposed to 3 mg/m^2^ PPF) but not when exposed 24 hours before a bloodmeal, challenging the idea that treating bednets would be a successful intervention for this species.

*Culex quinquefasciatus* is another important disease vector responsible for the transmission of *Wucheria bancrofti* (lymphatic filariasis), and arboviruses like Western equine encephalitis virus, St Louis encephalitis virus and West Nile virus [34,35]. It is also an abundant nuisance mosquito in many tropical and subtropical areas [36,37]. Conflicting reports arise from two studies that evaluate the impact of PPF on exposed *Cx. quinquefasciatus*. Whilst Mosqueira and colleagues [38] reported both a reduction in the number of eggs laid and hatchings in *Cx. quinquefasciatus* exposed 24–36 hours before a blood meal to an insecticidal paint formulation that contained PPF, Ngufor and others [33] found that exposure of *Cx. quinquefasciatus* to PPF-treated nets while seeking a blood meal had no effect on the reproductive capacity.

Whilst the sterilizing effect of PPF on adult mosquito vectors is by itself an important aspect to study for developing novel vector control strategies, it is also likely that it affects the potential of a female to transfer the insecticide to a larval habitat. The major challenge in the development of such an auto-dissemination approach is therefore to find the best timing and strategy to expose female mosquitoes to PPF to ensure that a large quantity of the insecticide gets picked up and transferred to an aquatic habitat. The best knowledge we have of the behaviour of *An. gambiae* s.s. is its indoor host-seeking and resting behaviour associated with the need for a blood meal [39-42]. Consequently, contaminating females during this time period would be the easiest e.g. exposing females to treated resting sites [17] or bednets [30], however, this timing might coincide with sterilizing effects and affect the ability to transfer PPF. Another challenge of the auto-dissemination approach for malaria control is the low density of adult anophelines in comparison to the surface area of the aquatic habitats [43]. To increase the amount of PPF transferred to *An. gambiae* s.l. larval habitats, other co-habiting mosquito species i.e. *Culex* mosquitoes [44-46] might also be targeted for transfer, especially since their immature stages are frequently of a greater density [43,47-49].

Here, we explored the effect of PPF exposure at different points in time before and after a blood-meal on the egg-laying and hatching of eggs in *An. gambiae* s.s. and *Cx. quiquefasciatus* and how this affects their ability to transfer PPF to a breeding site. We had the following hypotheses: (1) PPF exposure of adult *An. gambiae* s.s. and *Cx. quinquefasciatus* affects their ability to lay eggs and the number of offspring hatched from eggs laid, (2) the impact is largest when exposure takes place around blood-feeding time (3) the concentration of PPF transferred by a single female is very low requiring a large number of females to transfer lethal concentrations (LC_99_) (4) sterile females do not transfer PPF and (5) for auto-dissemination of PPF females need to be exposed not more than 24 hours prior to oviposition.

## Methods

### Mosquitoes

The study was carried at the International Centre of Insect Physiology and Ecology, Thomas Odhiambo Campus (*icipe*-TOC) located in Mbita, along the shores of Lake Victoria, Western Kenya (geographic coordinates 0° 26’ 06.19” S, 34° 12’ 53.13”E; altitude 1,137 m above sea level) with larvae and pupae of *An. gambiae* s.s. and *Cx. quinquefasciatus* obtained from the *icipe*-TOC’s insectary. Larvae were reared in round plastic tubs (diameter 0.6 m) filled with 5 litres of water (height approximately 5 cm) from Lake Victoria filtered through a charcoal-sand filter. Mosquito larvae were fed with Tetramin© Baby Fish food twice daily. Mosquito larvae were reared at ambient climate and light conditions in a netting-screened greenhouse with a temperature of 25-28ºC, relative humidity of 68-75% and a natural 12 hours of dark and 12 hours of light cycle. Pupae were collected from tubs and transferred into holding cages measuring 30x30x30 cm covered with mosquito netting. Adults were provided with 6% glucose solution *ad libitum*. Mosquitoes of both species were provided with a single blood -meal when they were three days old; *An. gambiae* s.s. fed on a human arm for 20 minutes whilst *Cx. quinquefasciatus* were fed on a rabbit for 20 minutes. The females of either species were maintained in cages with equal numbers of males of the same species at all times to increase the chances of insemination.

### Test insecticide

An experimental formulation of Sumilarv® dust containing 2% of PPF was provided by the manufacturer, Sumitomo Chemicals, Japan. Dust particles measured approximately 12 μm diameter. Sumilarv® is a registered trademark of Sumitomo Chemical Company.

### Exposing female mosquitoes to PPF

A suspension was prepared by mixing 0.25 g of the insecticide with 10 ml of acetone in a 100 ml glass beaker and vortexing on a shaker for 20 minutes. The inner surfaces of plastic jars (7.8 cm diameter, 9.2 cm height, 350 ml capacity) were coated by pipetting 150 μl of the suspension (0.075 mg active ingredient) into the jar. To ensure uniform coating of the base and side surfaces an additional 100 μl of acetone was added to the jar. The jar was then rolled several times on its base and side. The total surface area coated was approximately 0.028 m^2^ to give a concentration of 2.6 mg/m^2^ of active ingredient. A control jar of similar measurements was treated in a similar manner with acetone. Jars were left to air-dry for 30 minutes. New suspensions and jars were used for every treatment and replicate round.

**Figure 1:**
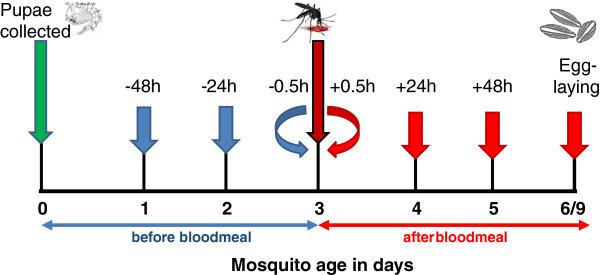
**Schematic diagram showing the pyriproxyfen-exposure times for *Anopheles gambiae *s.s. and *Culex quinquefasciatus *.** Blue arrows show treatment groups exposed before a bloodmeal, red arrows show treatment groups exposed after a bloodmeal. Control females were exposed to acetone at 0.5 hours before bloodmeal. Time of egg-laying was in *Anopheles gambiae* s.s. 72 hours after a bloodmeal (6 day old females) and in *Culex quinquefasciatus* 144 hours after a bloodmeal (9 day old females). All treatment groups and control were tested in parallel, 20 individual females at a time, repeated 4–5 times (rounds).

Female mosquitoes originating from the same batch of pupae per round were exposed to PPF at the following times in relation to when they bloodfed (Figure 1): (1) 48 hours before a blood meal (2) 24 hours before a blood meal (3) 0.5 hours before blood meal; and (4) 0.5 hours after a blood meal (5) 24 hours after a bloodmeal (6) 48 hours after a blood meal, and (7) on the day of egg-laying (72 hours after a blood meal in *An. gambiae* s.s. and 144 hours after a blood-meal in *Cx. quinquefasciatus*). Control females were exposed to acetone-only contaminated jars 0.5 hours before a bloodmeal.

Groups of 150 females per treatment per round were transferred to a PPF-contaminated jar covered with non-contaminated mosquito netting for 30 minutes. Most of the females rested on the plastic, but when a mosquito attempted to rest on the mosquito netting it was gently disturbed to rest on the contaminated surfaces of the jar. After exposure they were transferred into 30×30×30 cm cages per treatment group and an equal number of males added to maximize the chance of females mated at the time of experiment. Glucose solution (6%) was provided *ad libitum*. On the day of experiment (see below) 20 gravid females per treatment were selected from their holding cages.

### Measuring the effect of PPF exposure on a females ability to lay eggs and the ability of the egg to hatch

Oviposition experiments were implemented 72 hours after a blood meal with *An. gambiae* s.s. and 144 hours after a blood meal with *Cx. quinquefasciatus* based on the different egg maturation times. For each experimental round and treatment 20 gravid females were selected individually from their holding cage and transferred to netting covered cages of 15x15x15 cm at 18:00 h. A single female was introduced into a cage that contained a glass cup (7 cm diameter) filled with 100 ml of non-chlorinated tap water for oviposition. *Anopheles gambiae* s.s. females exposed to PPF 72 hours after a bloodmeal and *Cx. quinquefasciatus* exposed to PPF 144 hours after a bloodmeal were transferred directly from the exposure jar into the experimental cages containing an oviposition cup. Mosquitoes were left to lay eggs overnight. The following morning the presence of eggs or egg rafts was recorded, and in the case of *An. gambiae* s.s. the number of eggs counted, and then transferred into separate 300 ml plastic cups filled with 100 ml non-chlorinated tap water. The number of larvae that hatched from eggs laid by individual females was recorded.

Five rounds of the experiment were carried out with *An. gambiae* s.s. (5 × 20 replicates/treatment) and four rounds with *Cx. quinquefasciatus* (4 × 20 replicates/treatment) on separate dates. Therefore, in total 100 individual *An. gambiae* s.s. and 80 individual *Cx. quinquefasciatus* females were tested in each treatment arm.

### Assessment of delayed egg-laying in PPF-exposed *An. gambaie* s.s

To assess whether PPF exposure caused delayed egg-laying in female *An. gambiae* s.s., tests were conducted with females exposed to PPF: (1) 24 hours before a blood meal, (2) 0.5 hours before a blood meal, (3) 0.5 hours after a bloodmeal and (4) 24 hours after a blood meal. These were compared to a control group of females that were exposed to acetone 0.5 hours before a blood meal. Females were prepared as described above and provided with oviposition cups 72 hours after a blood meal and left to lay eggs overnight. The following morning the presence and number of eggs laid by each female was recorded. Thereafter, fresh oviposition cups were provided in all cages with the same mosquitoes maintained in the cages with 6% glucose solution *ad libitum*. The oviposition cup was left in the cage for a further two days to determine if mosquitoes would lay eggs. These tests were conducted in three rounds on separate dates with each round containing 20 replicates of each treatment and the control group. Thus in total 60 individual mosquitoes per treatment and control group were tested.

### Analyses of transfer of PPF by adult *An. gambiae* s.s. and *Cx. quinquefasciatus* to the water in the oviposition cups

To evaluate whether *An. gambiae* s.s. and *Cx. quinquefasciatus* transferred PPF to the water, 10 insectary-reared late instar *An. gambiae* s.s. larvae were introduced into all the oviposition cups in the morning after the removal of the eggs. For that, larvae were randomly collected from rearing tubs in the larval insectary to ensure that equal sizes of larvae were used in the experimental cups [50]. The larvae were monitored daily for mortality or pupation. During the monitoring period mosquito larvae were fed on fish food (Tetramin© Baby) daily. This was done by wetting a blunt toothpick in non-chlorinated tap water followed by dipping less than 1 mm of toothpick into the larval food. The toothpick was then dipped onto the surface of the test water. Pupae were transferred into a separate glass cup with approximately 50 ml of non-chlorinated tap water and the cup covered with mosquito netting to prevent any escape of emerged adults. Pupae were monitored for adult emergence.

### Statistical analyses

Generalized estimating equations (GEE) were used to analyze the data. The experimental round was included as repeated measure. Proportions were analyzed by fitting a binomial distribution with logit link function and counts analyzed by fitting a negative binomial distribution with log link function. An exchangeable correlation matrix was assumed. Treatment group was included as the fixed factor in the models with the control group as reference. All means (proportion or counts) per treatment and their 95% confidence intervals (CIs) were modelled as the exponential of the parameter estimates for models with no intercept included. Multiple comparisons of treatments were also calculated based on the model parameter estimates. Abbott’s formula [51] was used to calculate proportion reductions in egg-laying responses, egg-hatching success and emergence of adults from larvae introduced in the different treatment groups taking the natural response/mortality of the control group into account.

### Ethical considerations

Ethical approval for this study was obtained from the Kenya Medical Research Institute’s Ethical Review Committee (Protocol no. 422).

## Results

### Effect of PPF exposure on a females’ ability to lay eggs and the ability of the egg to hatch

PPF exposure affected *An. gambiae* s.s. and *Cx. quinquefasciatus* egg-laying as early as 48 hours prior and up to 24 hours after a blood meal in *An. gambiae* s.s. and 48 hours after a blood meal in *Cx. quinquefasciatus* (Table 1). However, the proportion of females laying was only reduced by approximately one third when exposed to PPF 48 hours before a blood meal compared to the control group (Table 1). The highest reduction due to the treatments in both species was roughly 60%, which was achieved by PPF exposure between 24 hours before and 24 hours after a blood meal in *An. gambiae* s.s. and between 24 hours before and 0.5 hours after a blood meal in *Cx. quinquefasciatus*. In *An. gambiae* s.s., the odds of laying as compared to not laying in the control was 3.3:1, whilst the odds of laying versus not laying was on average 0.45:1 in females exposed to PPF 24 hours before until 24 hours after a blood-meal. Hence, compared to the control the odds of laying was 7–8 times reduced (OR 0.12-0.15) when *An. gambiae* s.s. were exposed to PPF 24 hours before and up to 24 hours after a bloodmeal. Similarly, the odds of laying in *Cx. quinquefasciatus* was 4–9 times reduced (OR 0.11-0.25) when females were exposed to PPF between 24 hours before and 24 hours after a blood meal. Late contamination of *An. gambiae* s.s. with PPF at 48 hours and 72 hours after a blood meal and of *Cx. quinquefasciatus* at 144 hours after a blood meal did not affect the proportion of females laying eggs (Table 1).

**Table 1:** Effect of pyriproxyfen (PPF) exposure on the proportion of females laying eggs

**Exposure time to PPF in relation to blood meal**	**Proportion that laid eggs (95% CI)**	**Proportion reduction in laying (95% CI)**	**Odds ratio (95% CI)**	**p-value**
***Anopheles gambiae*** s.s.*				
72 hours after	0.80 (0.73-0.87)	0	1.20 (0.68-2.14)	0.460
48 hours after	0.80 (0.75-0.85)	0	1.21 (0.78-1.88)	0.390
24 hours after	0.33 (0.24-0.43)	0.56 (0.48-0.66)	0.15 (0.08-0.29)	<0.001
0.5 hours after	0.31 (0.23-0.41)	0.59 (0.50-0.68)	0.14 (0.05-0.34)	<0.001
0.5 hours before	0.33 (0.24-0.43)	0.57 (0.48-0.66)	0.15 (0.11-0.20)	<0.001
24 hours before	0.29 (0.21-0.39)	0.62 (0.52-0.70)	0.12 (0.07-0.21)	<0.001
48 hours before	0.52 (0.42-0.62)	0.32 (0.24-0.41)	0.32 (0.18-0.60)	<0.001
Control	0.76 (0.71-0.82)	-	1	
***Culex quinquefasciatus*** **				
144 hours after	0.68 (0.58-0.76)	0.05 (0.02-0.10)	0.87 (0.67-1.12)	0.450
48 hours after	0.58 (0.47-0.78)	0.19 (0.14-0.27)	0.48 (0.34-0.68)	0.020
24 hours after	0.41 (0.31-0.52)	0.43 (0.33-0.52)	0.25 (0.14-0.43)	<0.001
0.5 hours after	0.24 (0.16-0.34)	0.66 (0.56-0.75)	0.11 (0.08-0.16)	<0.001
0.5 hours before	0.29 (0.20-0.40)	0.59 (0.48-0.69)	0.14 (0.09-0.20)	<0.001
24 hours before	0.31 (0.22-0.42)	0.56 (0.46-0.66)	0.16 (0.10-0.27)	<0.001
48 hours before	0.46 (0.36-0.57)	0.36 (0.26-0.44)	0.31 (0.18-0.51)	<0.001
Control	0.72 (0.65-0.78)	-	1	

*Egg-laying took place 72 hours after bloodmeal.

**Egg-laying took place 144 hours after bloodmeal.

Of those few *An. gambiae* s.s. that laid eggs, the mean number of eggs laid per female was reduced by 21-36% compared to the control females if exposure to PPF occurred between 24 hours before and 24 hours after a blood meal, whilst the numbers were similar to the control when exposure occurred 48 hours and 72 hours after a blood-meal (Table 2).

**Table 2:** **Mean number of eggs laid by unexposed and pyriproxyfen-exposed *An. gambiae *** **s.s**

**Exposure time to PPF in relation to blood meal**	Mean no. of eggs* (95% CI)	**Odds ratio (95% CI)**	**p-value**
72 hours after	49.4 (45.5-53.6)^a,c**^	0.97 (0.86-1.09)	0.580
48 hours after	49.4 (46.4-52.6)^a,c^	0.97 (0.88-1.07)	0.520
24 hours after	37.8 (32.3-44.2)^b,c^	0.74 (0.62-0.90)	0.002
0.5 hours after	32.9 (27.9-38.7)^b^	0.64 (0.53-0.79)	<0.001
0.5 hours before	40.0 (34.2-46.8)^a,b^	0.78 (0.65-0.95)	0.010
24 hours before	40.3 (34.1-47.6)^a,b^	0.79 (0.65-0.97)	0.019
48 hours before	45.0 (39.8-51.0)^a,b^	0.88 (0.76-1.03)	0.110
Control	51.1 (47.9-54.4)^a^	1	

*Only females that laid eggs were included in analysis.

**Values without letters in common differ significantly (p < 0.05) in mean number of eggs laid.

It was 13–20 times less likely for an *An. gambiae* s.s. egg to hatch into a larva (OR 0.05-0.08) when the mother was exposed to PPF between 24 hours before and 24 hours after blood feeding (Table 3). However, there was no difference in egg hatching in eggs laid by *An. gambiae* s.s. exposed close to oviposition time with those laid by control females (Tables 3). The impact of PPF exposure on the mean number of larvae that successfully hatched from an egg raft of *Cx. quinquefasciatus* was only moderately reduced by 1.3-1.7 times compared to egg hatching in the control (Table 3).

**Table 3:** Effect of pyriproxyfen (PPF) exposure of female mosquito on hatching of her eggs

***Anopheles gambiae s.s.***				
**Exposure time to PPF in relation to blood meal**	Mean proportion eggs hatched* (95% CI)	**Proportion reduction in hatched larvae (95% CI)**	**Odds ratio (95% CI)**	**p-value**
72 hours after	0.86 (0.85-0.87)	0	0.99 (0.80-1.23)	0.910
48 hours after	0.84 (0.82-0.85)	0	0.99 (0.82-1.19)	0.910
24 hours after	0.22 (0.19-0.24)	0.73 (0.71-0.77)	0.06 (0.05-0.09)	<0.001
0.5 hours after	0.19 (0.17-0.23)	0.77 (0.73-0.80)	0.05 (0.03-0.07)	<0.001
0.5 hours before	0.21 (0.18-0.23)	0.75 (0.72-0.78)	0.06 (0.05-0.08)	<0.001
24 hours before	0.24 (0.22-0.27)	0.71 (0.68-0.73)	0.08 (0.06-0.11)	<0.001
48 hours before	0.54 (0.51-0.56)	0.35 (0.34-0.38)	0.27 (0.22-0.34)	<0.001
Control	0.84 (0.83-0.85)	-	1	
***Culex quinquefasciatus***				
**Exposure time to PPF in relation to blood meal**	Mean no. of larvae hatched per egg raft ** (95% CI)	**Proportion reduction in hatched larvae (95% CI)**	**Odds ratio (95% CI)**	**p-value**
144 hours after	76.4 (75.3-77.5)	0.07 (0.05-0.09)	0.94 (0.89-0.98)	0.008
48 hours after	66.0 (60.6-72.0)	0.19 (0.16-0.21)	0.81 (0.77-0.86)	<0.001
24 hours after	66.4 (63.7-69.2)	0.18 (0.14-0.22)	0.82 (0.77-0.87)	<0.001
0.5 hours after	67.8 (59.9-76.8)	0.17 (0.10-0.23)	0.83 (0.70-0.99)	0.035
0.5 hours before	61.9 (56.2-68.2)	0.24 (0.18-0.29)	0.76 (0.69-0.83)	<0.001
24 hours before	72.9 (65.0-81.7)	0.10 (0.5-0.16)	0.90 (0.76-1.03)	0.130
48 hours before	51.2 (49.4-53.0)	0.37 (0.34-0.40)	0.63 (0.60-0.66)	<0.001
Control	81.4 (76.6-86.6)	-	1	

*Eggs were counted for *An. gambiae s.s.* and the proportion that hatched calculated.

**The number of eggs per egg raft was not counted. Comparisons are made between mean numbers of larvae per egg raft.

PPF exposure did not induce any significant delays in egg-laying. The exposure either sterilized the female so that she did not lay at all, or she laid 72 hours after the last blood meal like unexposed control females (Table 4).

**Table 4:** **Evaluation of delayed egg-laying in *An. gambiae *** **s.s. due to pyriproxyfen exposure**

**Exposure time to PPF in relation to blood meal**	**Females exposed**	**Females laying eggs 72 hrs after blood meal**	**Females laying eggs later than 72 hrs after blood meal**	**Had the female laid eggs before?**
24 hours after	60	14	0	_
0.5 hours after	60	23	1	No
0.5 hours before	60	24	0	_
24 hours before	60	17	1	No
Control	60	49	2	No

### Transfer of PPF by adult *An. gambiae* s.s. and *Cx. quinquefasciatus* to the water in the oviposition cups

Transfer of PPF to the oviposition substrate and consequent emergence inhibition of introduced late instar *An. gambiae s.s.* larvae was assessed separately for the following two groups: (1) oviposition substrates originating from females that laid eggs; and (2) oviposition substrates originating from females that did not lay eggs.

**Figure 2:**
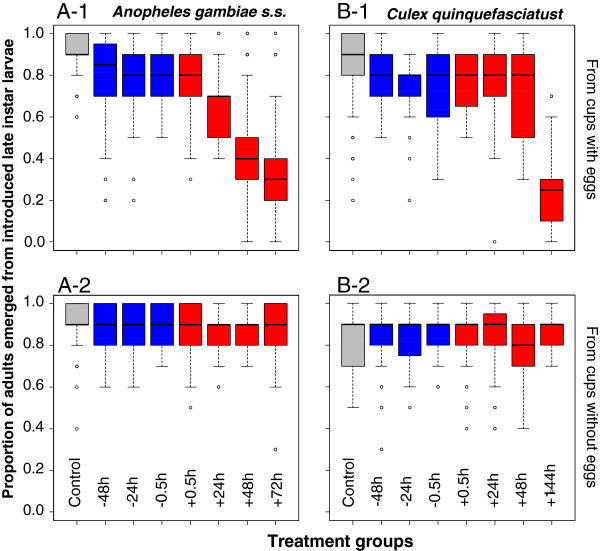
**Median adult emergence rates from late instar larvae introduced into oviposition cups.** Results for pyriproxifen exposed *Anopheles gambiae***(A)** and *Culex quinquefasciatus***(B)** from cups in which eggs were laid (1) and for cups in which no eggs were laid (2). Blue box plots show treatment groups exposed before a bloodmeal, red box plots show treatment groups exposed after a bloodmeal.

Emergence was inhibited from all treatments compared to the control when females laid eggs. However, the reduction was very low with 13-28% emergence inhibition from cups that were visited by *An. gambiae* s.s. females exposed to PPF between 48 hours before to 24 hours after a blood meal and 6-19% emergence inhibition from cups that were visited by *Cx. quinquefasciatus* females that were exposed between 48 hours before to 48 hours after a blood meal (Table 5, Figure 2). Biologically significant emergence inhibition was only achieved when females were exposed to PPF very close to oviposition time i.e. 52-65% from treatments with *An. gambiae* s.s. exposed 48 hours to 72 hours after a blood meal and 71% from treatments with *Cx. quinquefasciatus* exposed 144 hours after a blood meal.

**Table 5:** Adult emergence from late instar larvae introduced into oviposition substrates

**Exposure time to PPF in relation to blood meal**	**Mean adults emerged (95% CI)**	**Proportion emergence inhibition (95% CI)**	**Odds ratio (95% CI)**	**p-value**
***Anopheles gambiae*** **s.s.**				
**Females that laid eggs**				
72 hours after	0.32 (0.29-0.35)	0.65 (0.62-0.68)	0.04 (0.03-0.05)	<0.001
48 hours after	0.44 (0.41-0.46)	0.52 (0.51-0.54)	0.07 (0.05-0.09)	<0.001
24 hours after	0.66 (0.60-0.71)	0.28 (0.24-0.33)	0.18 (0.14-0.25)	<0.001
0.5 hours after	0.75 (0.70-0.80)	0.18 (0.14-0.22)	0.31 (0.19-0.51)	<0.001
0.5 hours before	0.78 (0.73-0.82)	0.15 (0.21-0.19)	0.36 (0.26-0.50)	<0.001
24 hours before	0.79 (0.74-0.83)	0.14 (0.11-0.18)	0.38 (0.25-0.58)	<0.001
48 hours before	0.80 (0.76-0.83)	0.13 (0.10-0.16)	0.38 (0.17-0.83)	0.015
Control	0.92 (0.90-0.93)		1	
**Females that did not lay eggs**				
72 hours after	0.88 (0.84-0.92)	-	0.80 (0.48-1.33)	0.380
48 hours after	0.87 (0.84-0.90)	-	0.69 (0.47-1.03)	0.070
24 hours after	0.86 (0.83-0.88)	-	0.62 (0.41-0.94)	0.020
0.5 hours after	0.86 (0.84-0.89)	-	0.67 (0.38-1.17)	0.160
0.5 hours before	0.88 (0.86-0.90)	-	0.79 (0.50-1.23)	0.290
24 hours before	0.90 (0.88-0.92)	-	0.96 (0.57-1.61)	0.870
48 hours before	0.89 (0.86-0.91)	-	0.82 (0.56-1.21)	0.320
Control	0.90 (0.88-0.93)	-	1	
***Culex quinquefasciatus***				
**Females that laid eggs**				
144 hours after	0.25 (0.22-0.29)	0.71 (0.67-0.74)	0.07 (0.06-0.09)	<0.001
48 hours after	0.70 (0.66-0.74)	0.19 (0.16-0.21)	0.28 (0.15-0.53)	<0.001
24 hours after	0.78 (0.73-0.82)	0.09 (0.07-0.13)	0.39 (0.29-0.54)	<0.001
0.5 hours after	0.76 (0.70-0.82)	0.12 (0.07-0.16)	0.37 (0.30-0.46)	<0.001
0.5 hours before	0.74 (0.68-0.79)	0.14 (0.10-0.19)	0.32 (0.16-0.65)	0.002
24 hours before	0.71 (0.65-0.76)	0.17 (0.14-0.23)	0.28 (0.23-0.36)	<0.001
48 hours before	0.81 (0.76-0.84)	0.06 (0.04-0.10)	0.46 (0.34-0.62)	<0.001
Control	0.86 (0.84-0.88)		1	
**Females that did not lay eggs**				
144 hours after	0.84 (0.79-0.87)	-	1.17 (0.94-1.45)	0.170
48 hours after	0.78 (0.73-0.82)	-	0.78 (0.61-0.99)	0.038
24 hours after	0.83 (0.80-0.86)	-	1.08 (0.60-1.96)	0.790
0.5 hours after	0.84 (0.81-0.87)	-	1.14 (0.90-1.45)	0.270
0.5 hours before	0.86 (0.83-0.88)	-	1.31 (0.88-1.95)	0.180
24 hours before	0.84 (0.80-0.87)	-	1.13 (0.69-1.83)	0.640
48 hours before	0.84 (0.80-0.87)	-	1.14 (0.83-1.57)	0.410
Control	0.82 (0.78-0.85)		1	

Conversely, when females did not lay eggs in the provided oviposition cup, emergence of introduced larvae was the same as in the control for all treatments and both species (Table 5, Figure 2).

## Discussion

Our study confirms a strong sterilizing effect of PPF on both *An. gambiae* s.s. and *Cx. quinquefasciatus* when females were exposed within 24 hours before or after a blood meal. Moreover, in our simple system we demonstrated that gravid females can transfer lethal concentrations of PPF to oviposition sites. However, our results suggest that for the use in an auto-dissemination approach females of both species would need to be exposed to PPF when already gravid so that sufficient PPF can be delivered to aquatic habitats.

The effect of PPF exposure on *An. gambiae* s.s. was three fold as it reduced the proportion of females laying eggs, the number of eggs laid and the number of eggs that successfully hatched into larvae when females were exposed to 2.6 mg/m^2^ PPF between 24 hours before and 24 hours after a blood meal. However, the main effect of PPF exposure on *Cx. quinquefasciatus* during the same time interval was only in reducing the number of females laying eggs.

Overall, the number of offspring produced by females exposed to PPF 24 hours before to 24 hours after a blood meal was reduced between 91-94% in *An. gambiae* s.s. and 60-75% in *Cx. quinquefasciatus* compared to control females. The differences in sterilization between the two mosquito species might be explained by the larger size of *Cx. quinquefasciatus* relative to *An. gambiae* s.s. and their different ability to metabolize insecticides [52]. Thus, it is likely that larger concentrations of PPF are required to increase the impact of topical application on *Culex* mosquitoes.

The dependence of exposure time to PPF on reducing egg laying and hatching in mosquitoes has been shown in other studies [13,17,32], however, the reported results are not consistent. For instance while Itoh and colleagues [13] observed a reduction in number of eggs laid by *Ae. aegypti* exposed to PPF on the same day of bloodmeal, Sihuincha and colleagues [15] reported that exposure of the same mosquito species at a similar point in time did not affect the number of eggs laid. Only few studies have been done on the effect of PPF on egg-laying and hatching in *Anopheles* mosquitoes with contrasting findings. Aiku and colleagues [30] reported that *An. stephensi* exposed to bednets treated with 2% PPF at 24 hours after blood meal were as likely to lay and laid similar numbers of eggs as control mosquitoes but eggs were less likely to hatch. However, Miller [31] found that exposure of the same mosquito species to bednets treated with 0.5 mg PPF/m^2^ at the time of blood meal caused a reduction in number of eggs laid. These differences on the effect of PPF might be explained by the variations in PPF dosages used in the separate studies and the characteristics of surfaces onto which PPF is applied [32,38].

Our study confirms the observation of Ohashi and colleagues [32] that exposure of laboratory reared *An. gambiae* s.s. females to PPF at comparable dosages before and after a blood meal significantly reduces the number of offspring produced from these females. A recent study by Ngufor and colleagues [33] also found complete sterilization in wild pyrethroid-resistant *An. gambiae* s.s. that came into contact with PPF treated nets while seeking a blood meal. Our observations extend their evidence by showing that the sterilizing effect can be achieved during a relatively large window of time between 24 hours before to 24 hours after a bloodmeal and at a relatively low concentration. Our results contrast, however, with those of Harris and colleagues [17] that showed for the sibling species *An. arabiensis* a sterilizing effect when exposure took place 24 hours after the blood meal but not 24 hours before the blood meal. Further studies might be warranted to explore the individual susceptibility of these closely related species further when aiming at developing intervention strategies targeting both sibling species by topical application at the same time.

Our study provides strong evidence that exposure of adult vectors, both anophelines and culicines to PPF can contribute significantly to reduce their population density. The sensitivity of both *An. gambiae* s.s. and *Cx. quinquefasciatus* to sterilization by PPF close to a blood meal presents an excellent opportunity to integrate PPF in insecticide-treated bednets, include PPF in indoor spays or wall paints to apply on inner surfaces of houses to reduce mosquitoes’ reproductive capacity as females seek a blood meal or as they rest indoors after taking a blood meal. This impact would be greatly enhanced when sterilization occurs in successive gonotrophic cycles in addition to reduced lifespan as previously shown for *An. gambiae* s.s. exposed to PPF-treated nets [32]. However, if both species should be targeted by the intervention, more research might be required to find the optimum dosages. Our findings on the sterilizing effect of PPF on *Cx. quinquefasciatus* confirm previous findings from a study on insecticidal paint containing PPF [38]. Yet, a recent experimental hut trial with wild *Cx. quinquefasciatus* could not demonstrate any impact of exposed to treated nets on this species [33]. Unfortunately, this study does not report the PPF dosage and one can only speculate that the larger size of the mosquito combined with a lower resting time on contaminated surfaces might be responsible for the differences between studies.

We were able to demonstrate in principle that female *An. gambiae* s.s. and *Cx. quinquefasciatus* can transfer PPF from contaminated resting surfaces to aquatic substrates. This study demonstrated that the greatest adult emergence inhibition occurred when *Cx. quinquefasciatus* females were exposed to PPF immediately prior to oviposition. Thus targeting gravid *Culex* species at their resting sites would increase the amount of PPF transferred to aquatic habitats in which immature stages of *An. gambiae* s.l. develop. However, the longer period in the gonotrophic cycle of *Culex* relative to that of *An. gambiae* s.s. presents a challenge in using *Cx. quinquefasciatus* or other *Culex* species for auto-dissemination. Whilst *An. gambiae* s.s. took 72 hours (3 days) after a blood meal to lay eggs, *Cx. quinquefasciatus* females laid eggs only 144 hours (6 days) after a blood meal. Studies have described the gonotrophic cycle in *An. gambiae* s.s to last 2–3 days [53,54], while that of *Cx. quinquefasciatus* and other *Culex* species lasts 3–6 days [55-58]. As shown in our study, this extended period increases the amount of PPF that this mosquito species will lose if exposure to the chemical is not done close to oviposition time. The loss of PPF overtime from body surfaces of mosquitoes has been explored in other studies [13,59].

The auto-dissemination technique has been successfully explored with *Aedes* mosquitoes in both laboratory and field settings [11-13,15,26,27,59,60]. Field studies have shown that *Ae. aegypti* and *Ae. albopictus* females can transfer PPF from limited contaminated resting sites to larval habitats to reduce adult emergence rates of developing larvae by 42-100% [11,12]. Three factors that are related to the oviposition behaviour of targeted *Aedes* mosquitoes contribute to the success of this strategy in the control of this mosquito species. First, *Aedes* mosquitoes utilize containers that hold small volumes of water as breeding habitats [61-63]. Second, laboratory assays indicate that 94% of *Ae. aegypti* distribute their eggs in up to seven oviposition cups in a single gonotrophic cycle, a phenomenon termed as skip-oviposition, [64] and field studies have shown that a relatively large number of females lay their eggs in a small oviposition container [65-68]. Third, PPF contamination in successful trials took place close to oviposition time [13,26,59]. These factors permit *Aedes* mosquitoes to accomplish several transfer events of PPF between contaminated surfaces and aquatic habitats to amplify adult emergence inhibition. *Aedes’* behaviour is in sharp contrast to that of *An. gambiae* s.l.. *Anopheles gambiae* s.l. colonizes natural habitats of varying size and stability [43,69,70] and is frequently found in extensive water bodies [71] with low larval densities per surface area [72,73]. Furthermore, molecular evidence of sibling relationships suggest that few females (average of 2–10 females) lay eggs in a typical larval habitat [74]. Although *An. gambiae* s.l. does skip-oviposit occasionally [75,76], it is not the norm in this species. A recent study [76] showed that approximately 20-30% of gravid females might choose more than one habitat to lay her eggs.

To our knowledge this is the first report of the potential use of the disease vectors, *An. gambiae* s.s. and *Cx. quinquefasciatus* for use in auto-dissemination of PPF to aquatic substrates to inhibit adult emergence. In the present study significantly higher emergence inhibition rates were recorded in oviposition cups where PPF-exposed female mosquitoes laid eggs compared to the controls. However, sterilized females that were exposed to PPF between 24 hours before and after a bloodmeal did not transfer sufficient PPF to water to cause biologically important emergent inhibition rates. There are two possible explanations for this phenomenon. First, sterile females have less or no mature eggs to lay [77,78] and therefore have little urge to visit aquatic substrates. Second, chemical analysis by high performance liquid chromatography (HPLC) reveal that early exposure of mosquitoes to PPF results in loss of greater amounts of the chemical before oviposition time [13].

Our study suggests that for *An. gambiae* s.s. and *Cx. quinquefasciatus* to optimally auto-disseminate PPF exposure must take place close to oviposition. However, even when both species were exposed that late only 65% and 71% emergence inhibition was achieved in oviposition substrates in which *An. gambiae* s.s. and *Cx. quinquefasciatus* laid eggs, respectively. Considering the small volume of water (100 ml) in a small oviposition cup of 0.004 m^2^ used here, it is estimated that two females of either species exposed to PPF immediately prior to oviposition would be required to transfer sufficient PPF to cause complete emergence inhibition in such a small habitat. This suggests that hundreds of mosquitoes would be required to transfer lethal concentrations to 1 m^2^ of habitat and the majority of natural habitats exceed this size [43]. This suggests that the auto-dissemination is less likely to be effective for control of *Anopheles* mosquitoes in the more difficult field situations than it is for *Aedes* control or would at least require PPF formulations with much higher percentage of the active ingredient than the 2% tested here. Further studies are needed to understand the behaviour of gravid mosquitoes as they leave the houses (or other feeding and resting locations) to lay eggs. This would help to gain knowledge of the outdoor resting surfaces of gravid *An. gambiae* s.l. to serve as potential auto-dissemination stations. Species-specific oviposition attractants might be used to lure gravid females to the auto-dissemination stations to pick up lethal doses of PPF for transfer to uncontaminated aquatic habitats [79,80].

## Conclusion

*Anopheles gambiae* s.s. and *Cx. quinquefasciatus* are highly affected by topical application of PPF reducing their viable offspring by 90% and 70%, respectively, when exposed to 2.6 mg/m^2^ one day before to one day after a blood meal. The time interval of greatest susceptibility is excellent for use on PPF treated materials and indoor sprays and paints on resting surfaces and could provide a significant contribution to malaria control by suppressing the vector population. Importantly, it presents a promising opportunity for integrated control of different vectors and nuisance mosquitoes. It is considered that the integration of PPF in available insecticides would help in the management of resistance to pyrethroids [33,81]. However, sterilized females are unlikely to visit an oviposition site and therefore do not transfer lethal concentrations of PPF to aquatic habitats. This suggests that for successful auto-dissemination the optimum time for contamination is close to oviposition, which requires further studies of the species’ resting behaviour after blood meals.

## Competing interests

Sumitomo Chemicals, Japan, provided the experimental formulation of the insecticide for this study free of charge. Nevertheless, neither the manufacturer nor any of the funders of this work had any role in the design, analysis or interpretation of the results, nor in the drafting of the manuscript.

## Authors’ contributions

UF and SWL conceived the idea for this research. OM, SWL and UF developed the experimental design and protocols. OM implemented the experiments, analysed the data and drafted the manuscript. All authors contributed to the final draft, read and approved the manuscript.
